# Toll‐like receptor 4: A promising therapeutic target for pneumonia caused by Gram‐negative bacteria

**DOI:** 10.1111/jcmm.14529

**Published:** 2019-07-27

**Authors:** Junying Ding, Qingquan Liu

**Affiliations:** ^1^ Beijing Key Laboratory of Basic Research with Traditional Chinese Medicine on Infectious Diseases Beijing China; ^2^ Beijing Hospital of Traditional Chinese Medicine Capital Medical University Beijing China; ^3^ Beijing Institute of Traditional Chinese Medicine Beijing China

**Keywords:** Gram‐negative bacterium, pneumonia, TLR4, Traditional Chinese Medicine

## Abstract

Gram‐negative bacteria (GNB) emerge as important pathogens causing pulmonary infection, which can develop into sepsis due to bacterial resistance to antibiotics. GNB pneumonia poses a huge social and economic burden all over the world. During GNB infection in the lung, Toll‐like receptor 4 (TLR4) can form a complex with MD2 and CD14 after recognizing lipopolysaccharide of GNB, initiate the MyD88‐ and TRIF‐dependent signalling pathways and stimulate host non‐specific immune response. In this review, we summarize recent progress in our understanding of the role of TLR4 in GNB pneumonia. The latest experimental results, especially in TLR4 knockout animals, suggest a promising potential of targeting TLR4 signalling pathway for the treatment of GNB pneumonia. Furthermore, we highlight the benefits of Traditional Chinese Medicine as novel candidates for the therapy of GNB pneumonia due to the modulation of TLR4 signalling pathway. Finally, we discuss the promise and challenge in the development of TLR4‐based drugs for GNB pneumonia.

## 1 INTRODUCTION

*Streptococcus pneumoniae* is a main cause of pneumonia, followed by other Gram‐positive bacteria such as *Staphylococcus aureus* and *Bacillus anthracis*. However, recent epidemiological study showed increasing incidence of pneumonia caused by Gram‐negative bacteria (GNB), and the most common is *Pseudomonas aeruginosa*, followed by *Klebsiella pneumoniae, Escherichia coli, Haemophilus influenzae*, *Bordetella pertussis* and *Moraxella catarrhalis*. Pneumonia caused by GNB is difficult to treat due to their antibiotic‐resistant characteristic. For example, *P aeruginosa* showed the resistance to most antimicrobials including ceftazidime, meropenem and piperacillin/tazobactam. *K pneumoniae* showed high rate of beta‐lactam resistance, including resistance to third‐generation cephalosporins and carbapenems.^1^ Moreover, pneumonia can lead to sepsis in immunocompromised hosts, which remains one of the major causes of death. The incidence and mortality rate of sepsis keep rising worldwide, especially in low‐ and middle‐income countries.^2^

Toll‐like receptor 4 (TLR4) can identify exogenous pathogens by binding to lipopolysaccharide (LPS) of GNB, stimulate the production of antimicrobial peptides and induce the non‐specific immune responses such as the activation of nuclear factor‐kB (NF‐kB) pathway in the macrophage.^3^ The activation of TLR4 by LPS is mediated by the interactions between LPS and several other proteins including LPS binding protein (LBP), the myeloid differentiation antigen (MD2), cluster of differentiation 14 (CD14) and TLR4. Finally, the activated complex LPS/MD2/TLR4 initiates the intracellular signalling pathway.^3^ TLR4 antibodies, inhibitors or antagonists which can affect the acetylation, dimerization or/and the recognition of ligands or receptors on TLR4 may inhibit the activation of downstream signalling, suggesting a strategy for pneumonia therapy via targeting TLR4 signalling. Especially, recent studies have shown that pneumonia can be treated with Traditional Chinese Medicine (TCM) via targeting TLR4.^4^ In the new era of antibiotic‐resistant bacteria,^5^ it is necessary to explore TCM for the treatment of GNB pneumonia based on the pivot role of TLR4 in infectious pneumonia.

## 2 GNB PNEUMONIA

Antibiotic‐resistant GNB infections become the leading causes of death caused by infectious pneumonia. Especially, uncontrolled inflammatory response to GNB infection is associated with high morbidity and mortality, which can turn pneumonia into sepsis due to the antibiotic resistance.

**Figure 1:**
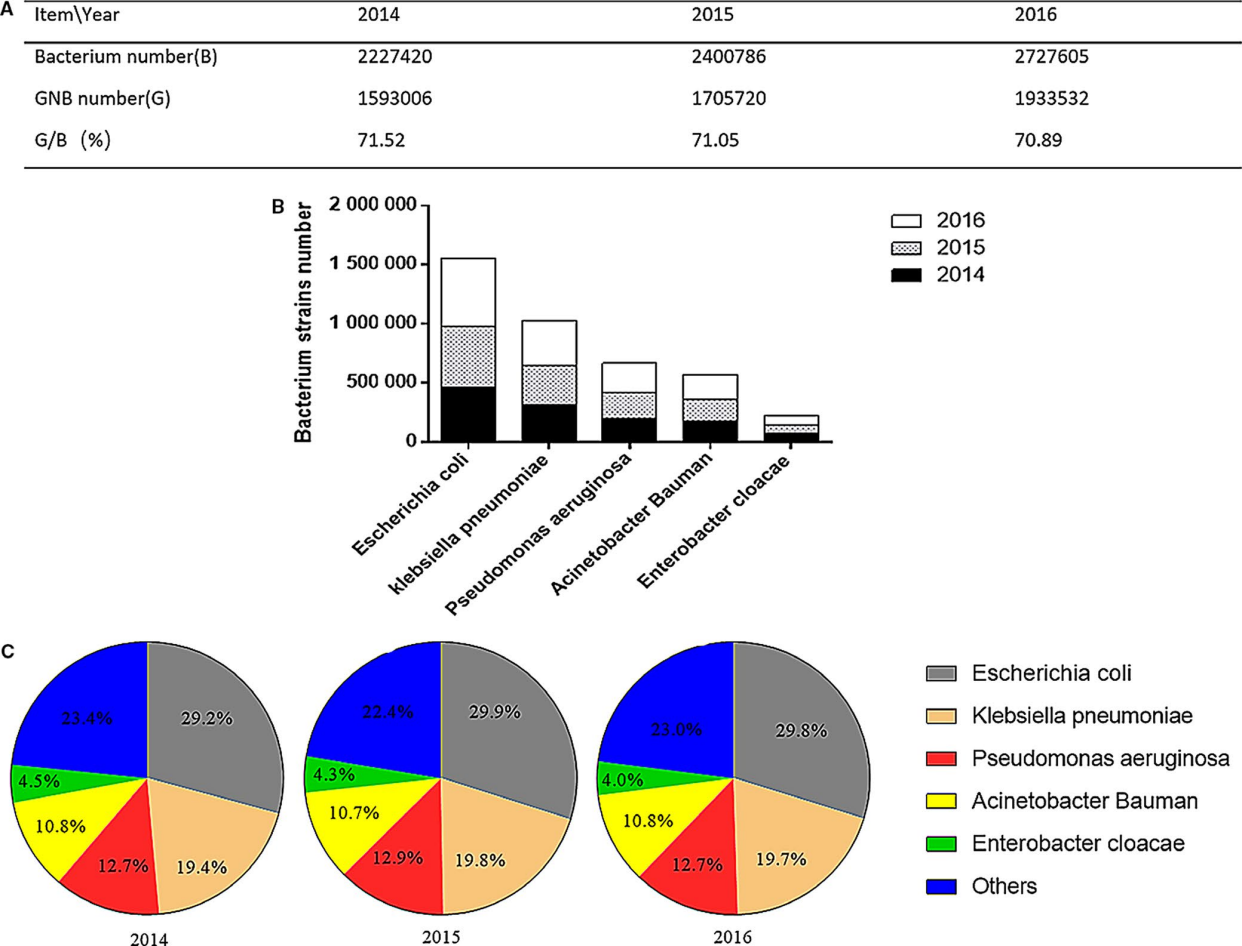
The number and proportion of GNB and top 5 GNB strains isolated from clinical patients in China during 2014‐2016. (A), The number and proportion of GNB isolated. (B), The number of top 5 GNB strains isolated. (C), The proportion of top 5 GNB strains isolated

Pneumonia resulting from GNB is a leading cause of mortality and morbidity with a rise in the prevalence of early‐onset ventilator‐associated and community‐acquired pneumonia. It was reported that bacterial antibiotic resistance could cause more than 25 000 deaths every year in Europe.^6^ In China, the prevalence of infectious diseases has been increasing yearly. From 2014 to 2016, the number of bacteria isolated from clinical cases, especially antibiotic‐resistant GNB, has kept increasing (Figure 1).^7^, ^8^, ^9^ Given the emergence of antibiotic‐resistant bacteria, it is urgent to develop new strategies to treat pneumonia by antibiotic‐resistant GNB.^10^, ^11^, ^12^

## 3 LPS ACTIVATES TLR4 SIGNALLING

Toll‐like receptor 4 plays a crucial role in mediating innate immune responses to infections in pneumonia, especially to GNB infection. LPS in the outer membrane of GNB can initiate the activation of TLR4 signalling.^3^ TLR4, MD2 and CD14 form a trimeric receptor complex through recognizing LPS.^13^ LPS‐mediated activation of TLR4/MD2 signalling plays a key role in the development and maintenance of beneficial host defence response 14 ^.^

### 3.1 Structure of tlr4

Toll‐like receptor 4 is the first identified member of TLR family.^15^ TLR4 is a transmembrane protein characterized by an extracellular domain containing leucine‐rich repeats (LRRs) where the MD‐2 molecule is associated, and a cytoplasmic tail harbouring a conserved region known as Toll/IL‐1 receptor (TIR) domain.^16^ The extracellular domain is responsible for ligand binding, receptor dimerization and initiation of intracellular signalling, whereas the intracellular domain shares a significant sequence and structural homology with the interleukin‐1 receptor (IL‐1R) family.^16^

### 3.2 Recognition of lps by tlr4

The complex crystal structure helps explain why LPS structural properties are ideal for TLR4 signalling activation. LPS has six lipid chains, five of which are completely submerged inside the pocket in MD‐2, whereas the sixth chain is exposed to the surface of MD‐2 and forms the hydrophobic interaction interface together with hydrophobic surface residues of MD‐2.^17^ After binding LPS, the TIR domain of TLR4 interacts with the TIR domain of myeloid differentiation factor 88 (MyD88), in conjunction with another TIR containing adaptor protein MyD88 adaptor like (MAL). Mutations of the TIR domains can abolish this interaction, suggesting that TIR domains are crucial to the formation of TLR4 signalling complex.^18^

## 4 MYD88‐ AND TRIF‐DEPENDENT SIGNALLING PATHWAYS IN PNEUMONIA

**Figure 2:**
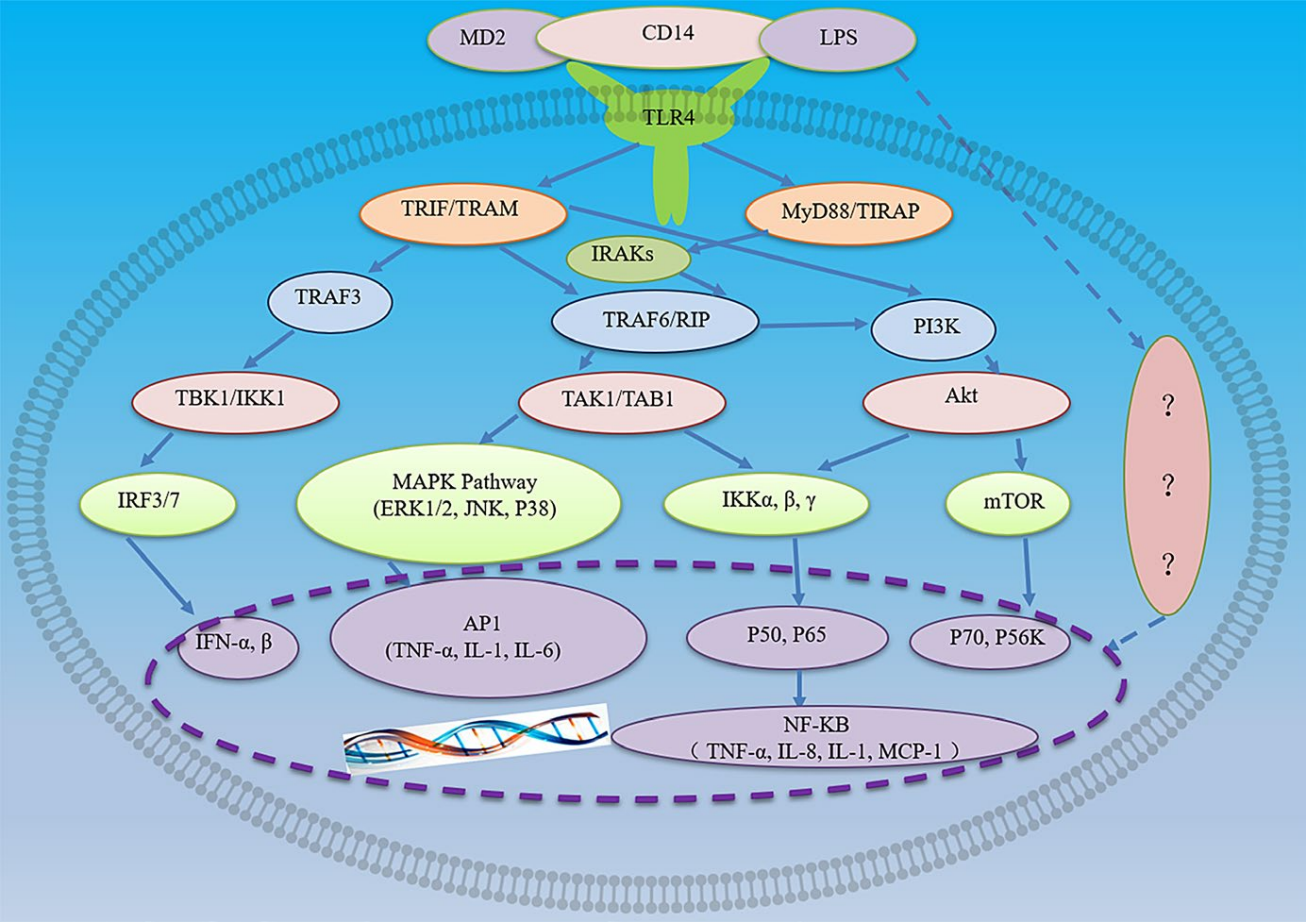
LPS/TLR4 signalling pathway. LPS binds to LBP and forms a complex with MD‐2/TLR4. Intracellular LPS/TLR4 signalling can be transduced through either MyD88‐dependent or MyD88‐independent TRIF/TRAM pathways, which mediate the transactivation of pro‐inflammatory cytokine

Both MyD88‐ and TRIF‐dependent pathways are implicated in TLR4‐mediated lung injury in pneumonia (Figure 2). The signalling pathways triggered by TLR4 engage adaptors that are recruited by TIR/TIR domain interactions, including MyD88, TIR domain‐containing adaptor protein (TIRAP, also known as MAL), TIR domain‐containing adaptor inducing interferon IFN‐β (TRIF) and TRIF‐related adaptor molecule (TRAM).

TIRAP services as a bridge for the recruitment of MyD88 to TLR4. MyD88 is essential to signal transduction through TLR4 and is involved in the activation of NF‐kB and mitogen‐activated protein kinases (MAPKs) and subsequent regulation of pro‐inflammatory gene expression.^19^ MyD88 consists of an N‐terminal death domain DD and a C‐terminal Toll/IL‐1 receptor (TIR) domain, separated by an intermediate linker region. Upon ligands binding, TLR/IL‐1Rs dimerize, followed by the recruitment of MyD88 through TIR domains. Then, through DD and TIR domains, MyD88 oligomerizes and interacts with the DDs of interleukin‐1 receptor‐associated kinases (IRAKs), leading to the formation of a multimeric complex myddosome.^20^ Myddosome formation results in the activation of MAPK signalling pathways, such as stress kinase p38 and extracellular signal‐regulated kinases 1 and 2 (ERK1/2), as well as the activation of transcription factors such as NF‐B and activator protein 1.

TcpB, a TIR domain‐containing protein produced by *Brucella melitensis*, can interact with MAL, MyD88 and TLR4. However, no interaction is observed between TcpB and TRAM or IRAK‐2, demonstrating that MAL‐dependent signalling pathway is the specific target for TcpB. Furthermore, competition assays demonstrated that TcpB can disrupt TLR4‐MAL interaction, but could not interfere with MAL‐MyD88 interaction.^21^ These results provide new insights into the mechanism of immunomodulation of TLR signalling by bacterial TIR domain‐containing protein at the structural level.

TcpB mimics MAL properties to bind to phosphoinositides in the plasma membrane through its N‐terminal domain and competes with MAL for the interaction with MyD88.^16^ However, Sengupta et al demonstrated that TcpB interacts with MAL but not with MyD88, and TcpB does not interfere with the interaction between MAL and MyD88.^22^ Site‐directed mutagenesis analysis showed that residues Glu^183^, Ser^244^ and Arg^288^ in the TIR domain are required for MyD88 homo‐dimerization, and mutations on these residues suppress the recruitment of IRAK1/4 and NF‐B activation. Importantly, overexpression of MyD88 mini‐proteins comprising the Glu183 residue competes with the homo‐dimerization of endogenous MyD88 protein and impairs TLR signalling in immune cells.^23^ These findings suggest that a novel region of MyD88 TIR domain is critical for TLR/IL‐1R signal transduction. WaaL mutant has been demonstrated to induce the activation of NF‐kB and the secretion of IL‐8 in wild‐type but not in MyD88 knockdown cells.^24^ Furthermore, in TLR4 knockdown model, both receptors of TLR4 contribute to waaL‐induced cell activation.^24^ Taken together, waaL mutant‐induced activation of NF‐kB and secretion of IL‐8 is mediated by TLR4‐MyD88 signalling pathway.

MyD88 aggregation signal is conveyed to IL‐1 receptor kinase (IRAK) through an interaction between the death domain of MyD88 and IRAK. The phosphorylation of the signalling kinases eventually activates transcription factors NF‐kB and activator protein 1. The activation of TLR4 can stimulate both MyD88‐dependent and MyD88‐independent pathways. MyD88‐independent pathway can induce the expression of IFN‐inducible genes, such as IP‐10 and glucocorticoid‐attenuated response gene. Through the recruitment of adaptor molecules, MyD88‐independent pathway leads to the late‐phase activation of NF‐κB. On the other hand, TRIF pathway of TLR4 activates interferon response factors, a family of transcription factors, to produce and secrete type I interferons.^25^, ^26^

TRIF initiates MyD88‐independent activation of IFN regulatory factor 3 (IRF3) and late NF‐kB activation, leading to the production of type I IFNs and the expression of IFN‐inducible genes. TRIF is recruited to the cytoplasmic domain of TLR3, which bridges TRIF to TLR4 through TRAM. Sterile a‐ and armadillo‐motif containing protein (SARM) is a negative regulator of TLR4 signalling pathway, which interacts with TRIF and suppresses the induction of TRIF‐dependent genes. NF‐kB is a key transcriptional activator that mediates immune response to bacterial and viral infection. Under normal conditions, NF‐κB is associated with members of the cytoplasmic IκB family of inhibitor proteins. Upon exposure to the stimuli such as LPS, receptor‐mediated phosphorylation of IκB is triggered, and then, phosphorylated IκB undergoes ubiquitination and proteasomal degradation. Meanwhile, NF‐κB dimer dissociates from its inhibitory protein IkB and shuttles into the nucleus, where it binds to specific response elements of target genes to enhance the transcription of target genes, such as inducible nitric oxide synthase (iNOS) and IL‐1β. Therefore, NF‐κB complex is a key mediator for LPS‐induced activation of transcription of cytokine genes.

Interestingly, a study showed that LPS pre‐conditioning redirected TLR4 singling via TRIF‐IRF3 pathway but not MyD88 pathway.^27^ The suppression of NF‐κB activity is repressed in LPS pre‐conditioning mice, whereas the production of pro‐inflammatory cytokine does not vary, suggesting that other signalling cascades are involved in the production of pro‐inflammatory cytokine.

## 5 THE ROLE OF TLR4 IN PNEUMONIA

It was reported that TLR4 expression at mRNA and protein levels significantly increased at around 2 to 6 hours after intracerebral haemorrhage, peaked at day 3, declined at day 5, while remained elevated compared with baseline even on day 7.^28^ In another study, exposure to LPS can enhance TLR4 mRNA expression after 1 hour in autologous human alveolar macrophages and monocytes, with a subsequent decrease in TLR4 mRNA level after 24 hours,^29^ suggesting that LPS can differentially affect TLR4 abundancy in alveolar macrophages. Chalk et al recently found that patients who developed pneumonia post‐operatively had decreased TLR4 expression in alveolar macrophages, suggesting that local cell‐mediated immunosuppression might be a risk factor for post‐operative pneumonia.^30^ TLR4 expression in alveolar macrophages in acute respiratory distress syndrome (ARDS) is suppressed, even after ex vivo stimulation to LPS.^30^

Toll‐like receptor 4‐deficient mice are valuable to study the role of TLR4 in pneumonia in vivo. Recently, several studies have highlighted the inflammatory role of TLR4 in pneumonia models. During lung infections, IL‐27 priming enhanced LPS‐induced production of IL‐6 and IL‐8 in lung fibroblasts via promoting TLR4 expression.^31^ Sansing et al demonstrated that TLR4 activation led to detrimental inflammatory response.^32^ However, in perihematomal inflammation, TLR4 signalling is not involved in transcriptional regulation of pro‐inflammatory cytokines.

It was reported that TLR4/MyD88‐triggered myeloid‐derived suppressor cells (MDSCs) help resolve inflammation during bacterial pneumonia.^33^ The cytoadherence of *M pneumoniae* induces inflammatory responses in macrophages through TLR4, and the response can be inhibited by TLR4 inhibitor VIPER.^34^ These results were confirmed in TLR2 and TLR4 double‐knockout (DKO) mice.^34^ In addition, TLR2/4 DKO mice were more susceptible to *K pneumoniae* infection than single TLR2 KO or TLR4 KO mice, suggesting that TLR2 and TLR4 play cooperative role in lung innate immune responses during *K pneumoniae* infection.^35^ However, *B pseudomallei* LPS signalling occurs solely through TLR4 in murine, whereas TLR2 plays an additional role in human.^36^

The patients who develop post‐operative pneumonia show a trend of significant reduction in TLR4 expression compared with those without pneumonia. Similarly, TLR4‐deficient mice had impaired survival with higher bacterial loads and diminished production of inflammatory mediators, indicating that TLR4 signalling is required to induce a protective immune response to common GNB.^37^ Based on these reports, the dual role of TLR4 in infectious pneumonia seems to be managed by the inhibitory or stimulatory factors for a balance. Nevertheless, TLR4 remains a potential target for inhibiting undesired inflammatory responses.

## 6 TCM TARGETING TLR4 AS POTENTIAL DRUG FOR PNEUMONIA

Due to the challenge from antibiotic‐resistant bacteria, it is very urgent for us to explore new treatment of pneumonia with TCM which will not cause antibiotic resistance. At present, various promising synthetic and plant‐derived strategies are being tested. Houttuynia can affect TLR4 expression directly or indirectly.^38^ Emodin inhibits influenza viral pneumonia, by inhibiting IAV‐induced activation of TLR4, MAPK and NF‐kB pathways and activating Nrf2 signalling.^39^ Ugonin M is a unique flavonoid isolated from Helminthostachys zeylanica, and it suppresses the production of pro‐inflammatory molecules such as nitric oxide, IL‐1, TNF‐α and IL‐6. Moreover, Ugonin M inhibits not only NF‐κB and MAPK activation but also TLR4 protein expression.^4^ These findings demonstrate that Ugonin M might exert efficacy on LPS‐induced lung infection and is the most effective component of *H *zeylanica used in pneumonia therapy. LianQinJieDu decoction (LQJD) is a Chinese traditional medicine used for respiratory tract infections, and its main ingredients are berberine, astragalus and scutellaria. LQJD was found to decrease LPS‐induced high body temperature, inflammatory cytokines expression level and lung injuries as well as to block the activation of TLR4/NF‐κBp65 signalling in lung tissue.^40^ *Astragalus membranaceus* and *Salvia miltiorrhiza* are well‐known Chinese traditional medicine, and a recent study reported that they had protective and therapeutic effects on LPS‐induced lung inflammation through modulating TLR‐4/NF‐κB signalling pathways.^41^ Sodium houttuyfonate (SH) is the active compound of Houttuynia plant, and it markedly attenuates pulmonary inflammation induced by LPS. The anti‐inflammatory effect of SH is associated with the inhibition of TLR4/NF‐κB activation through MyD88‐dependent pathway. Therefore, SH shows promise to treat pneumonia.^42^

## 7 VALIDATION OF TLR4 AS A PROMISING THERAPEUTIC TARGET FOR GNB PNEUMONIA

In recent years, with the development of genomics, structure biology and bioinformatics, increasing evidence validates that TLR4 is crucially involved in the pathogenesis of GNB pneumonia and emerges as a promising therapeutic target for GNB pneumonia.

A meta‐analysis showed that TLR4 A299G polymorphism was significantly associated with the susceptibility to pneumonia.^43^ It was reported that LPS O‐polysaccharide and T2SS mutant‐induced responses depended on TLR4‐MyD88 activation, suggesting LPS O‐polysaccharide and PulA T2SS as potential targets for designing antimicrobials.^24^ In addition, TLR4 mutant mice were more susceptible to butylated hydroxytoluene (BHT)‐induced pneumonia than TLR4 wild‐type mice. The distinct innate immune cell populations in TLR4 wild‐type mice were different from those in TLR4‐mutant model following BHT treatment.^44^ Computational analysis revealed that MD‐2 and NAMPT/PBEF were essential for LPS to induce TLR4 activation, but MD‐2 binding to TLR4 alone was insufficient to initiate TLR4 signalling.^45^

The missense mutations D299G and T399I were associated with LPS hypo‐responsiveness and increased susceptibility to infection by GNB, and the underlying mechanism may be due to the fact that D299G polymorphism could damage the recruitment of MyD88 and TRIF to TLR4 and the subsequent activation of downstream signalling pathway.^46^ In addition, TLR4 plays a crucial role in the phagocytosis of GNB. A recent study reported that TLR4 sorting adapter TRAM forms a complex with Rab11 family interacting protein 2 to activate GTPases Rac1 and Cdc42, and promote the phagocytosis of GNB.^47^ Collectively, these data validate the crucial role of TLR4 in the pathogenesis of GNB pneumonia.

## 8 CONCLUSIONS AND PERSPECTIVES

Based on all the above discussion, it is undoubted that TLR4 plays an important role in GNB pneumonia. TLR4 signalling pathway involved in lung inflammatory injury is composed of ligands, TLR4 itself, receptor and its downstream pathways, including adaptor proteins MyD88 and TRIF and transcription factors such as NF‐κB.^48^, ^49^ Therefore, TLR4 ligands and receptors are all promising targets for developing effective treatment of pneumonia. A variety of TLR4 antagonists has been identified (summarized in Table 1). Future studies are needed to investigate the interaction between TLR4 and these molecules in the initiation and development of GNB pneumonia.

**Table 1:** TLR4 antagonists and their function in TLR4 signalling pathway

Antagonist	Action target	Function
Curumin	Inhibiting TLR4 homodimerization	Blocking TLR4 signalling pathway
6‐shogaol	Inhibiting TLR4 homodimerization	Blocking TLR4 signalling pathway
Isoliquiritigenin	Inhibiting TLR4 homodimerization	Blocking TLR4 signalling pathway
TLR4 antibody	Blocking hemin‐induced microglial activation	Blocking TLR4 signalling pathway
Novimmune	TLR4‐blocking mAb	Blocking TLR4 signalling pathway
1A6	TLR4‐blocking mAb	Reducing inflammation responses
UT12	Promoting neutrophil recruitment	Augmenting innate immune responses
RGZPPARγ agonist）	Downregulating TLR4 expression	Reducing inflammation responses
PPAR γ	Suppressing proliferation and induced apoptosis	Inhibiting TLR4 signalling pathway
TRIF	Inducing IFN‐γ	Mediating antibacterial defences
NOX2	Co‐expression with TLR4	Regulating proinflammatory TLR4 signalling
Monophosphoryl lipid A	Restore antigen presentation	Dampening inflammatory lung lesions
AnxA2	Activating the TRAM‐dependent endosomal signalling	Negative regulation of inflammatory responses
EPCP1‐2	Downregulating TLR4 expression	Inhibiting macrophage proliferation Inhibiting TLR4 signalling
Oxymatrine	Suppressing TLR4 expression	Anti‐inflammatory responses
Ginkgolide B	Reducing neuronal cell apoptosis	Inhibiting TLR4 signalling pathway
Wogonoside and celastrol	Inhibiting MAPKs activation	Blocking TLR4‐mediated angiogenesis

Traditional Chinese Medicine has emerged as a novel approach to control GNB pneumonia due to the advantages of not causing antibiotic resistance. Recent studies have shown that TCM could target TLR4 signalling to achieve the efficacy to ameliorate or even eradicate GNB pneumonia. Therefore, it is important to explore TCM to develop new therapeutic intervention for pneumonia.^50^

However, it is important to notice that TLR4 signalling is involved in a variety of physiological and pathological processes in human body, and thus, the specificity of TLR4‐targeted therapy may be the biggest challenge in the clinical. Recent advances in crystal structure analysis of TLR4 and the development of better drug design tools may help address the challenge.

In summary, GNB pneumonia remains a serious threat to human health for the coming years. Appropriate screening and optimization of TLR4 agonists and antagonists, especially from TCM, would provide future therapeutics for GNB pneumonia.

## CONFLICT OF INTERESTS

All authors report no potential conflicts.

## AUTHOR CONTRIBUTION

Junying Ding wrote the manuscript and Qingquan Liu proof the manuscript.
